# Reports on Hospitals of the United Kingdom

**Published:** 1914-07-11

**Authors:** Henry Burdett


### REPORTS ON

### Hospitals of the United Kingdom.

By SIR HENRY BURDETT, K.C.B., K.C.V.O.

**SERIES III.**

### THE NORTH EVINGTON INFIRMARY, LEICESTER.

(Second Notice.)

### Report of the Guardians' Committee on Sir Henry Burdett's Report.

In our issue of May 30 last, pages 237-38, we published our Report upon this institution. At a meeting on May 19, 1914, the Leicester Guardians passed the following resolution: "That the best thanks of the Board be given to Sir Henry Burdett for his visit to the infirmary, and the Report which he had given thereon, and that the North Evington Infirmary Committee be requested to consider the Report in detail and report to the Board as to what steps they propose to adopt to rectify the defects pointed out therein." On June 11 the same Board resolved: "That Messrs. Gibson, Martin, Hancock, Dr. Crosby, Miss Irwin, and the Clerk draw up a reply to Sir Henry Burdett's Report."

Report of the Guardians' Committee.

At a meeting of the Guardians of the parish of Leicester, held on June 30, the following Report was adopted:

"Your Committee have carefully considered the questions arising out of the Report of Sir Henry Burdett upon his visit to the North Evington Infirmary, and by a detailed examination of the Minute Books have reviewed the work of the Guardians on these matters during the last few years.

"Your Committee beg to report that Sir Henry Burdett's recommendations have in the main been anticipated by resolutions adopted by the Guardians.

"The resolutions referred to are those dealing with:

"1. The erection of an operating room.

"2. The provision of increased nursing accommodation for additional nursing staff, and a recreation room.

"3. The appointment of a resident medical superintendent.

"The cause of any delay in carrying out the various proposals of the Guardians has been due to prospective legislation on the part of the Government.

"It is only right to say that, although the Local Government Board recommended delay with regard to the proposed buildings, their representatives were most sympathetic and helpful to the Guardians.

"With reference to the Maternity Building Sir Henry Burdett has been misinformed. Dr. Dodd has elsewhere pointed out that it is not, and never has been, an isolation unit for the reception of septic cases arising in the maternity department, and therefore it has not 'gradually been degraded from the purpose for which it was erected.'

"The Maternity Building was erected as an isolation block for infectious cases, but upon the Corporation agreeing to take charge of all infectious notifiable cases, it was turned into a maternity block, and it has been so used for years, septic cases being retained and isolated in the single rooms of Wards 15 and 16.

"The Guardians acknowledge, however, that Sir Henry has done valuable service in emphasising the necessity for the strictest care on the part of the medical staff respecting such cases.

"Finally, Sir Henry's opinion of the quality of the blankets used in the institution is at variance with that of the expert advisers of the Guardians, who state that they compare favourably with blankets that have come under their observation in other institutions."

### THE POSITION AT NORTH EVINGTON INFIRMARY.

The Committee do not fulfil their instructions by reporting "to the Board the steps they propose to adopt to rectify the defects pointed out." This is a most important fact. The report of the Committee is neither an answer to nor a contradiction of any matter raised in our Report on the North Evington Infirmary, as published in The Hospital of May 30 last, with the exception of one minor point—the blankets—and the criticism on what the Guardians term "the Maternity Building." As to the blankets we found them poor in quality—so poor that no such quality of blanket could be found, we are certain, in any voluntary hospital throughout the country. If these blankets "compare favourably" with blankets that have come under the observation of the Guardians' expert advisers in other institutions, all we can say is that the Poor-Law inspectors—if they were "the expert advisers"—charged with these matters are under a heavy responsibility, having regard to the fact that the quality of the blankets must minister materially to the comfort or suffering of the humblest people, who are quite defenceless in a matter of this kind.

Turning next to the Maternity Building, our Report stated: "We found . . . an admirable isolation unit for the reception of septic cases arising in the maternity department." The words "in the maternity department" should not have been used, as the Guardians' report rightly points out, seeing that the buildings were originally erected as an isolation block. With this slight correction we adhere absolutely to our statement in regard to the condition of the maternity department, as it in fact was when we inspected it in the company of the assistant medical officer, Dr. Fergus, on March 9, 1914. The unit has been gradually degraded from the purpose for which it was erected by putting there, as we stated, not only septic cases, but perfectly healthy maternity cases, whereby the health, and possibly the lives, of Leicester mothers must be imperilled. There was no proper isolation on the day of our visit, and in one ward containing two septic cases there was a vacant bed into which, we were informed, the next maternity case, however healthy, would be admitted in the ordinary course. We are glad, therefore, to note that the Guardians admit that the Report on this maternity block "has done valuable service in emphasising the necessity for the strictest care on the part of the medical staff respecting such cases." The Committee's report is valuable further in the sense that it inferentially confirms that the infirmary as planned contained an excellent maternity department and accommodation in the main building, with every facility for the treatment of non-septic maternity cases. The chief reason why non-septic cases were relegated to the infectious block was stated to be that the matron had ordered it. This statement surprised us, but we have now reason to believe the matron was made the responsible resident officer at the infirmary by order of the Local Government Board.

We can only hope that, in view of the Committee's finding, the Guardians will now be able to secure that the medical staff will not in future be superseded, but will be given the sole control over the admission and disposition of maternity cases at this infirmary, so that the strictest care to prevent the mingling of septic and healthy mothers may be once and for all guaranteed to all patients admitted to this institution.

The Committee omit altogether to refer to any other material portions of the Report and content themselves with the statement that "Sir Henry Burdett's recommendations have in the main been anticipated by resolutions adopted by the Guardians." We are perfectly certain we shall have the sympathy and support of every intelligent man and woman in Leicester when we declare that the mere passing of resolutions may be, as in the present instance, a danger, for it in no sense follows that they will produce practical results. For this reason, indeed, progress has been hampered at North Evington. The resolutions referred to had not behind them the necessary driving force. The present case is valuable as proving that no practical results can follow the mere passing of resolutions, however numerous they may be, or however worded.

We can well believe that the citizens of Leicester may have some difficulty in understanding the meaning of the Committee's report, and it may be well for us to try to interpret it by the recital of some facts of the first importance, to which possibly the unfruitful result of the Committee's report and labours may be attributed.

### Why the North Evington Infirmary is not an Up-to-date Hospital.

In our Report the following paragraph appeared: "The Leicester Board of Guardians are entrusted with the control of a magnificent group of buildings that only need the expenditure of a relatively small sum to make them into one of the most efficient and attractive hospitals in the Poor-Law service. All the difficulties, all the disagreements, all the criticism, and everything which is undesirable and regrettable in connection with the North Evington Infirmary in justice must be put down to the failure on the part of the Board of Guardians to realise the splendid opportunity they had. We have satisfied ourselves that everything which is regrettable and wrong at present can be speedily ended and removed by the appointment of a medical superintendent of ample experience and judgment, in whom should be vested the supreme control of the administration of the North Evington Infirmary."

The question arises, Why have the Board of Guardians failed to realise the splendid opportunity they have had during the last nine years? Their Committee plead that they have taken action, and that our recommendations in the main have been anticipated by resolutions adopted by the Guardians. Why is it that these resolutions have proved so barren in results? In examining this question we shall have to deal with the Local Government Board's action and attitude towards this infirmary. So long ago as 1911 the Guardians decided that it was desirable to extend the Poor-Law Infirmary. Again in 1913 they entered fully into the question of extensions, which were discussed, adopted, and ultimately resulted in proposals to extend the central administration block and, by the erection of a third storey, to provide additional accommodation for nurses. This further accommodation for nurses was, however, apparently so small as to be quite insufficient to accommodate the extra nurses required to provide for the nursing of 100 children and some 120 more patients, to say nothing of requisite additions to the nursing staff. It was decided to add two half-pavilions for ordinary sick cases—one for sixty-four males and one for sixty-four females, together with an operation theatre. The actual number of inmates thus provided for would have been some 128 adults and 106 children.

So far the Guardians appear to have thrice sent a deputation to the Local Government Board, one of which attended in July 1911, which resulted in postponement of action and a request for the preparation of a comprehensive scheme setting forth all the requirements and needs then apparent. Another deputation attended at Whitehall in May 1912, when the Local Government Board decided that the whole question should be deferred pending legislation. At the beginning of 1913 the needs of the infirmary appear to have impressed themselves upon the Local Government Board to such an extent that a conference was held in August of that year, when the proposals of the Guardians' Building Committee were fully gone into. Those proposals are, we believe, still under consideration, and this would appear to be fortunate, because the extensions in question are not calculated to make the North Evington Infirmary into an up-to-date hospital, but rather to degrade it still further by making it more and more resemble a scrap-heap for humanity. Such a waste of one of the finest hospitals under the Local Government Board would not fulfil the purposes which could alone justify the large expenditure which has been entailed by the erection of what we previously and properly described as an infirmary having many attractive features, most of which are excellent.

### A Condition of Crisis.

The North Evington Infirmary has, therefore, arrived at a condition of crisis which should seriously affect not only the future action of the Local Government Board, but the individual responsibility of each member of Parliament for Leicester. One is entitled to believe that the best interests of the humblest of the citizens of Leicester, though they may not necessarily have votes, should be considered by the leader of the Labour Party in Parliament as a matter of the first importance, which he cannot properly or at all neglect. We venture to put a perfectly straight case in regard to the present position of the North Evington Infirmary to Mr. Ramsay Macdonald, M.P. For nine years the results of a very large expenditure of ratepayers' money in the production of most excellent buildings for a modern up-to-date Poor-Law infirmary have been largely wasted, owing to the fact that the infirmary has never yet been organised as a hospital. Had it been intended originally merely to provide accommodation for dements, idiots, epileptics, and aged people of both sexes, there certainly could have been no justification for the large expenditure which was incurred in bricks and mortar. That fact in itself would be serious enough, but the passing of the National Insurance Act has made it even more serious still. Hospital accommodation in England is deficient already by quite 10,000 beds if it is to be sufficient to enable every insured person now requiring in-patient care to obtain it promptly and efficiently. The North Evington Infirmary, as our Report proved, could readily and inexpensively be made into an up-to-date modern hospital, and so immediately fulfil the purpose for which it was originally planned. The inhabitants of Leicester are clearly entitled to claim that this step should be forthwith taken, having regard to the great cost incurred.

The Guardians' Committee rightly plead in self-defence that they have anticipated the recommendations we ventured to make by resolutions. Why have those resolutions not proved effective? Simply because every proposal put forward, including that for an operation theatre made so long ago as 1911, has been postponed. Have these postponements at the suggestion of the Local Government Board been due to an intelligent anticipation by the Government authorities of the need for extra hospital accommodation which the passing of the National Insurance Act would engender? If so, we venture to point out that it is not right for the Local Government Board to prefer or to sanction further extensions to the North Evington Infirmary which will provide additional accommodation not for acute cases but for the feeble-minded and epileptics, for imbeciles, and even for patients who ought properly to be admitted and retained in an asylum. It is, indeed, contrary to the interests of the people and to public policy to pursue such a course any further. If Mr. Ramsay Macdonald desires to remain M.P. for Leicester he must face the position and act with promptitude. The urgent need of the moment is to secure that the North Evington Infirmary shall be restored as speedily as possible to its proper purpose—that of an up-to-date hospital dealing with cases of tuberculosis, labour, and acute disease. Economical considerations, justice to the ratepayers, the poor, and the Leicester Board of Guardians, to say nothing of the authorities at Whitehall, unite in support of such a statesmanlike policy.

